# The relationship between physical activity and depression in college students: the parallel mediating role of mindfulness and life satisfaction

**DOI:** 10.3389/fpubh.2025.1618840

**Published:** 2025-08-25

**Authors:** Anning Wang, Longjuan Shi, Tong Wang

**Affiliations:** ^1^College of Physical Education and Sports Science, Qufu Normal University, Qufu, Shandong, China; ^2^Chengdu Vocational University of the Arts, Chengdu, Sichuan, China; ^3^School of Sports Training, Chengdu Sports University, Chengdu, Sichuan, China

**Keywords:** depression, mindfulness, life satisfaction, physical activity, college student

## Abstract

**Objective:**

To explore the relationship that mindfulness and life satisfaction have in the process of alleviating depression through physical activity in Chinese universities and the mediating role of life satisfaction and mindfulness in this process.

**Methods:**

Participants were 508 college students (240 males, 268 females, mean age 19.90, SD = ±1.10). Psychosocial tests, including the Physical Activity Rating Scale (PARS-3), the Positive Attention Awareness Scale (MAAS), the College Student Life Satisfaction Scale (CSLSS), and the Patient Health Questionnaire-9 (PHQ-9), were completed. Correlations of variables were calculated using Pearson’s test. The mediation model was tested using the SPSS 26.0 PROCESS macro plug-in and regression bootstrap.

**Results:**

Physical activity showed a negative correlation with depression (*β* = −0.0045, *p* < 0.001), mindfulness and life satisfaction showed a correlation between physical activity and depression, and more importantly, physical activity was negatively correlated with depression through the parallel mediating effect of mindfulness and life satisfaction, with a total mediating effect of 53.54%.

**Conclusion:**

By increasing college students’ participation in physical activity, it helps to promote their mindfulness and life satisfaction, which is the key to reducing depression and promoting college students’ mental health.

## 1 Introduction

Depression is the most devastating mental illness and is one of the top 25 causes of burden worldwide (1). Depression is a subsyndrome involving symptoms of depression and negative mood (2, 3). Its main clinical manifestations are low mood, agitation, loss of interest, lack of pleasure, physical illness, sleep disturbance, loss of appetite, slow thinking, low self-esteem, and suicidal tendencies, which seriously interfere with the work and life of patients and their families. According to the World Health Organization, depression has become a common mental illness worldwide, with 280 million people suffering from varying degrees of depression, accounting for 4% of the global population (4). Depression is also the most prevalent psychological problem among college students (5). In previous studies, the prevalence of depression was higher in the college student population, with the highest prevalence in Brazilian college students (62.93%), followed by the United States (33%), China (28.9%), Pakistan (10.67%), and the lowest in Australia (7.9%) (6, 7). Studies have shown that social factors such as social development, increased competition, and educational reforms have exacerbated the incidence of depression among college students. In addition, lack of physical activity, leading to obesity and sedentary behavior, are also important reasons that exacerbate the incidence of depression (8). In China, with the increase in academic and employment pressures, the prevalence of depression is even higher among Chinese college students (9, 10). Depression causes Chinese college students to experience academic collapse (such as procrastination, avoidance, and decline in attention and memory), self-isolation, eating disorders, social anxiety, and other symptoms, which seriously affect the normal lives of college students. How to effectively reduce and improve the prevalence of depression is becoming a hot topic in Chinese academia. In recent years, participating in sports activities has been considered one of the effective ways to alleviate the prevalence of depression (11, 12).

Physical activity is defined as engaging in physical activity with a specific frequency, intensity, and duration (13). Physical activity reduces mental health problems such as depression, anxiety, and stress while being effective in preventing lifestyle diseases such as obesity, diabetes, cardiovascular disease, and hypertension. It affects a range of biological and psychosocial processes and have been shown to be involved in the pathophysiology of depression (14). Some studies have shown that high-intensity and moderate-intensity physical activity has significant health benefits in alleviating depression (15). There is a negative correlation between moderate to vigorous physical activity and depression (16). Conversely, depressed individuals tend to engage in less physical activity than non-depressed individuals (17). In addition, regular physical activity plays a crucial role in preventing mental illness in college students, especially those who face a variety of challenges (18). It has been confirmed in previous theoretical models and empirical studies that participation in physical activity significantly improves negative mood induced by stress or sedentary behavior, thereby reducing the prevalence of depression (19). Physical activity plays a key role in reducing the prevalence of depression and is considered an effective intervention that may help lower the incidence of depression. Additionally, college students have more opportunities to participate in physical activities on campus. Therefore, physical activity may be a more effective way for college students to alleviate symptoms of depression.

Mindfulness is a valuable way to reduce psychological distress and simultaneously promote subjective well-being. Originally made into a discipline of consciousness. Conceptualized by Walsh as a specific mental discipline to enhance the perception and awareness of emotions and experiences to achieve inner peace and freedom (20). Later defined as the non-judgmental awareness of its momentary experiences, including sensations, emotions, and bodily feelings (21). Mindfulness encourages individuals to take a neutral stance in observing their thoughts and experiences and prevents acceptance of the present moment (22). Furthermore, failure to cultivate mindfulness in a timely manner may lead to negative outcomes; for example, research has shown that individuals feel pain from neutral events they experience (23). Conversely, mindfulness promotes an existence-oriented lifestyle. The aid of mindfulness can help individuals reduce negative emotions such as stress, depression, and anxiety (24, 25). In addition, research has shown a positive correlation between mindfulness and resilience and life satisfaction (26). Low cost, easy access, and no side effects are key factors that make mindfulness more beneficial to college students than other methods. Based on this, mindfulness may be one effective way to improve life satisfaction and reduce depression and anxiety (27).

Life satisfaction significantly affects depression. It is a subjective judgment of a person’s quality of life and involves a comparison between an individual’s standard of living and their own expectations (28). By measuring the congruence between their current life, expected life, and actual life. It reflects a person’s psychological state. Existing research shows that life satisfaction has been shown to have a key role in predicting mental health problems. Researchers have analyzed the relationship between life satisfaction and various psychological problems, and it is not only associated with behaviors such as anxiety, suicidal tendencies, alcohol dependence, drug addiction, and Internet addiction but also significantly associated with depression (29). In addition, studies have shown that lower life satisfaction is associated with poor psychological states. Some studies have confirmed the positive correlation between mindfulness behaviors and life satisfaction (30). In summary, college students’ life satisfaction is strongly linked to their mental health, resilience, social relationships, and campus integration. Exploring the relationship between life satisfaction can further highlight the individual development and mental health of college students.

There are more studies that have explored the risk of depression, the factors that influence it, and how to alleviate it. And so far, engaging in physical activity, enhancing mindfulness, and increasing life satisfaction have been shown to be effective in reducing depression, suggesting an association between these factors (31–33). However, there is a lack of research in the college student population that specifically explores physical activity to reduce depression, especially in relation to mindfulness and life satisfaction. Whereas in positive psychology, mindfulness and life satisfaction are among the notable variables, relevant studies have highlighted their role in alleviating depression. This provides a theoretical basis for further research, especially in the context of Chinese society, where there may be differences in depression among college students across regions and ethnicities (34, 35). Therefore, the present study aimed to explore the relationship between physical activity and college students’ depression, to analyze the mediating roles of mindfulness and life satisfaction, and to provide a theoretical and empirical basis for reducing the level of depression among college students, as well as deepening the understanding of the psychological mechanisms of depression.

## 2 Theoretical foundations

### 2.1 Physical activity and depression

Based on the theoretical frameworks of exercise physiology and clinical psychology, the present study defined physical activity as energy-consuming body movements produced by skeletal muscle contraction, which encompasses structured exercise and unstructured daily behaviors. From the perspective of psychopathology, according to the Affective Response Hypothesis, regular physical activity can improve depression-related affective disorders by regulating limbic system function through neurobiochemical mechanisms (36), while the Social Withdrawal Theory emphasizes the role of exercise intervention in enhancing the efficacy of social connections and rebuilding interpersonal relationships (37). The social withdrawal theory emphasizes the therapeutic potential of exercise interventions in enhancing social bonding efficacy and reestablishing patterns of interpersonal interaction, which further supports the alleviation of depression and anxiety disorders. Regular exercise and consistent participation in physical activity during college have been emphasized to prevent and alleviate depression among college students. Existing research suggests that regular participation in sports can prevent or alleviate depression (38). In addition, a study on physical activity, sleep, and depression in college students indicated that rigorous physical activity and quality sleep are beneficial in helping and regulating depressive symptoms (39). Therefore, based on the foundation of the above studies, this study proposes Hypothesis 1: There is a negative relationship between physical activity and depression among college students.

### 2.2 The mediating role of mindfulness

Regarding mindfulness, it refers to the characteristic tendency of individuals to maintain awareness of the current moment in a non-reactive and non-judgmental way. According to the framework of Self-Determination Theory, mindfulness can increase an individual’s awareness of internal experiences and external environment (40). It is currently being studied extensively in a variety of contexts. These include the enhancement of academic performance, the reduction of anxiety, and the improvement of stress (41), in addition to confirmed links with structural brain networks (42). There are also more studies reporting the link between mindfulness and mental health. Studies have shown that mindful individuals have good emotional regulation and resilience. This helps individuals to alleviate their bad emotions and negative thoughts (43). On the contrary, individuals with low levels of mindfulness are prone to irrational thoughts and behaviors in response to stress (21), leading to an increased risk of developing depression (44). Based on existing research, this study proposes Hypothesis 2: Mindfulness mediates the relationship between physical activity and depression among college students.

### 2.3 The mediating role of life satisfaction

Life satisfaction is a hot topic in positive psychology research. It is an individual’s subjective evaluation of his or her overall quality of life, as well as a comprehensive indicator for evaluating an individual’s sense of well-being. With the continuous and in-depth development of positive psychology, people increasingly recognize life satisfaction as an important indicator of physical and mental health development. In the theoretical framework of sport psychology, physical activity is an effective means to improve life satisfaction (45). Notably, it has been shown that sleep quality and optimism increase life satisfaction in older adults, thereby reducing the risk of anxiety and depression. In addition, more studies have shown that improving life satisfaction can reduce depression levels in college student populations, which has been demonstrated in Korean and Malaysian college student populations (46, 47). Therefore, based on previous research, this study proposes Hypothesis 3: Life satisfaction mediates the relationship between physical activity and depression.

### 2.4 The effect of gender on the relationship between physical activity and depression

Research on mediating effects has been used to elucidate and explore the relationship between physical activity and depression, but this mechanism still suffers from the shortcoming of insufficient explanatory power, and such effects are more variable across different contexts. Therefore, this study draws on gender role social theory and other relevant empirical studies to introduce gender as a moderating variable to more effectively explore the relationship between physical activity and depression in different contexts. Existing research suggests that female college students have higher stress levels and are more likely to be emotionally unstable and feel stressed, which leads to their higher levels of depression (48). A cross-cultural context study similarly confirmed that female students reported significantly higher levels of drowsiness than male students, further adding to their increased levels of depression (49). Based on this, the present study synthesized evidence from previous studies and constructed a parallel mediation model to explore the relationship and mechanisms between physical activity and depression, using social role theory and self-determination theory as a theoretical framework. Specifically, this study first considered the mediating roles of mindfulness and life satisfaction in the relationship between physical activity and depression and tested the moderating role of gender in these mediating pathways, aiming to provide empirical support and theoretical guidance for college students to reduce and alleviate depression levels by participating in physical activity.

## 3 Materials and methods

### 3.1 Participants

Given that the research design and variables in this study differ from those in previous studies (50), *a priori* power analysis was conducted using G*Power software (51) to determine the appropriate sample size required for the parallel mediation effect model constructed for the test. Given that the model involves multiple mediation paths, we employed the linear multiple regression method from the F-test: The fixed model, R^2^ deviation from zero method, commonly used in parallel mediation effect studies, is particularly suitable for predicting overall effects before conducting bootstrapping tests (52). The effect size was set to *f* = 0.25, corresponding to f^2^ = 0.0625, based on Cohen’s (53) standard definition of a moderate effect. This effect size was chosen because moderate strength mediating effects are common in related behavioral science and psychology research and have a strong empirical foundation (54), thus providing robust theoretical and empirical support. This study sets the significance level *α* = 0.05 and the degree of certainty (1-*β*) = 0.95. Based on the above parameters, the minimum sample size required to test the model is approximately 210 participants to ensure sufficient statistical power for effective identification of the mediating effect when it truly exists.

This study employed convenience sampling to recruit 530 undergraduate students from two universities in Shandong Province, northern China, to participate in a questionnaire survey. Recruitment was conducted through a combination of classroom announcements and online questionnaire distribution. Prior to participation, all participants were briefly informed of the study’s objectives and signed written informed consent forms. Subsequently, participants completed an anonymous questionnaire covering basic demographic information, which also provided foundational data for subsequent follow-up studies.

The questionnaire was distributed and collected between January and April 2025. After excluding 22 invalid questionnaires (e.g., answered too quickly, inconsistent content, or logical contradictions), a total of 508 valid samples were obtained, including 240 males and 268 females, with an average age of 19.90 years (standard deviation = ±1.10) and an effective response rate of 95.84%. Detailed sample information is provided in Table 1.

**Table 1:** Descriptive statistics (*n* = 508).

Projects	Categories	Cases	Percentage
Gender	Male	240	47.24%
	Female	268	52.76%
Age	19	223	43.90%
	20	197	38.78%
	21	29	5.71%
	22	33	6.50%
	>22	26	5.12%
Student’s degree	Undergraduate	431	84.84%
	Bachelor’s degree	75	12.40%
	PhD	10	2.76%

This study adhered to the ethical principles of the 1964 Declaration of Helsinki and its subsequent revisions. The research protocol was approved by the Ethics Committee of the School of Physical Education, Qufu Normal University (Ethics Approval Number: QF2025002), and all participants signed informed consent forms.

### 3.2 Test tools

#### 3.2.1 Physical activity rating scale (PARS-3)

The Physical Activity Rating Scale (PARS-3) revised by Liang was used in this study (55). The scale determines the physical activity level based on the intensity, frequency, and duration of physical activity and categorizes the individual activity level into five grades and assesses them from 1 to 5. The total physical activity score is calculated as follows: intensity x (duration of exercise - 1) x frequency of exercise, with a score range of 0–100. The higher the score, the higher the level of physical activity, where a score ≤19 indicates a low level of activity, a score between 20 and 42 indicates a moderate level of activity, and ≥43 indicates a high level of activity. In this study, the Cronbach’s alpha coefficient for this scale was overall = 0.701.

#### 3.2.2 Positive attention awareness scale (MAAS)

The MAAS developed by Brown et al. was used in this study (56). The Chinese version of this scale has been validated for reliability (57). The scale consists of 15 items on a 5-point Likert scale ranging from “very consistent” to “very inconsistent.” An example sentence for one item is “I’m in the middle of something that I’m not paying attention to.” It is used to assess an individual’s attention and awareness of what is currently happening, with higher scores indicating higher levels of mindfulness and vice versa. In this study, the Cronbach’s alpha coefficient for this scale was overall = 0.916.

#### 3.2.3 College student life satisfaction scale (CSLSS)

In this study, the College Student Life Satisfaction Scale (CSLSS) revised by Wang Yuzhong was used (58), which was divided into 6 items of objective satisfaction (e.g., academic performance, relationship with friends, etc.) and subjective satisfaction (e.g., total satisfaction with one’s own life, etc.), with a 5-point Likert scale from very satisfied to very dissatisfied. In this study, Cronbach’s alpha coefficient for this scale was overall = 0.799.

#### 3.2.4 Depression scale

The Patient Health Questionnaire-9 (PHQ-9) was used in this study to assess the level of depression in the subjects (59). The PHQ-9 contains nine items from the Diagnostic and Statistical Manual of Mental Disorders. A 5-point Likert scale was used, ranging from strongly agree to strongly disagree, and example sentences for the items included “feeling frustrated or hopeless” and “not having any interest or pleasure in doing things,” with scores ranging from 0 to 27, with higher scores indicating more severe depressive symptoms. In this study, the Cronbach’s alpha coefficient for this scale was overall = 0.863.

### 3.3 Statistical analysis

**Figure 1:**
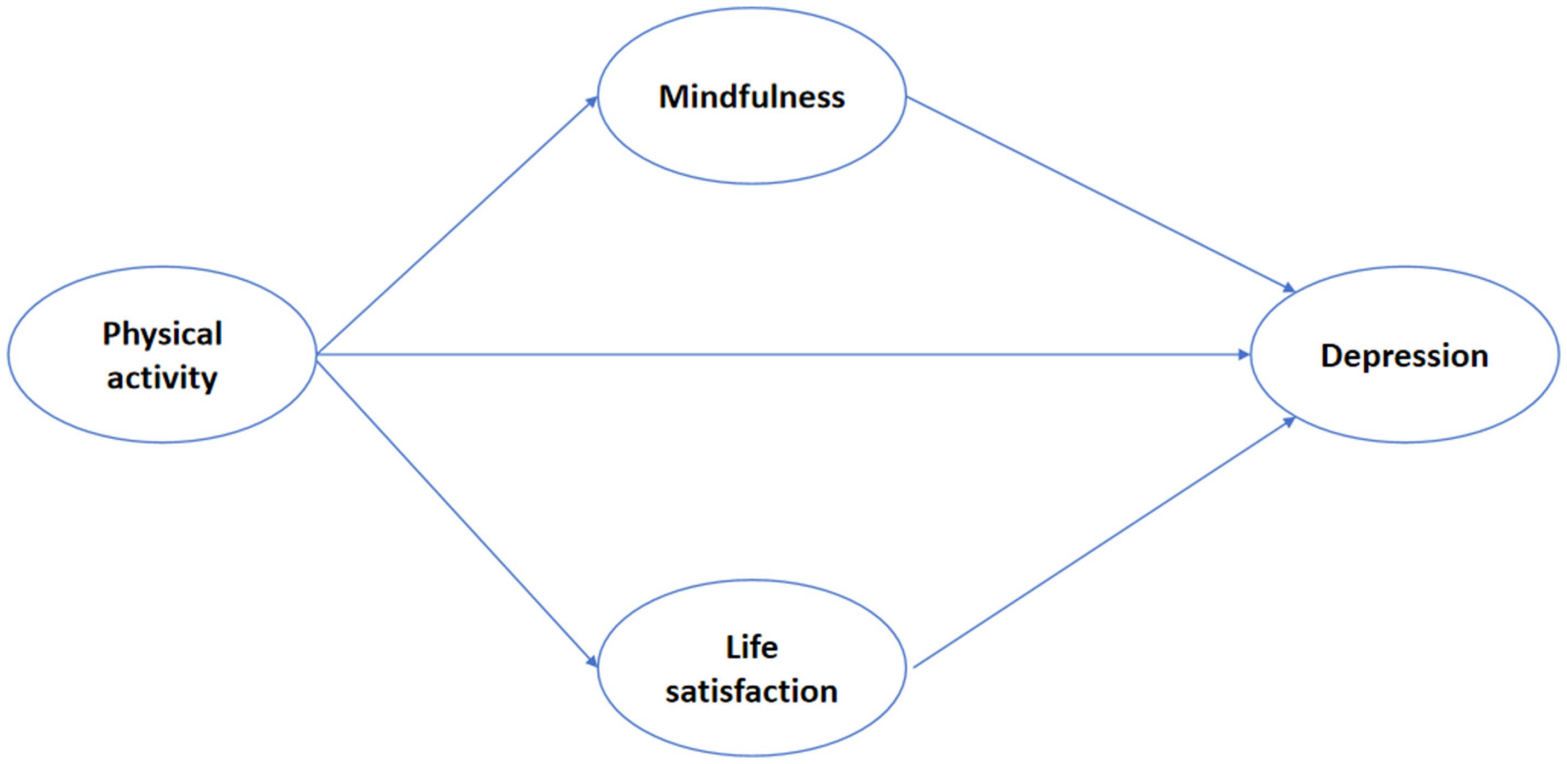
Hypothetical model of this study.

Data analysis was conducted using the SPSS statistical software package (version 26.0). Categorical variables were presented as frequencies (n) and percentages. This study employed analysis of covariance (ANCOVA), independent samples t-tests, and the Mann–Whitney U test, combined with Kolmogorov tests and normal distribution tests, to explore differences in physical activity, mindfulness, life satisfaction, and depression. To examine the potential mediating role of mindfulness and life satisfaction between physical activity and depression, this study utilized Model 4 from the SPSS PROCESS 4.0 plugin provided by Hayes (60) for analysis. In this model, physical activity serves as the independent variable, mindfulness and life satisfaction as mediating variables, and depression as the dependent variable (Figure 1).

In the parallel mediation model of this study, age and gender were included as covariates to control for their potential confounding effects on the independent variable, mediating variables, and dependent variable, and to further explore their potential influence on the overall model. This analysis aimed to examine the mediating effects of mindfulness and life satisfaction between physical activity and depression. Data analysis was conducted using SPSS software, with the Model 4 in the PROCESS macro used to test the mediating effects (60). Specifically, the 95% confidence intervals for indirect effects were constructed using the bias-corrected Bootstrap method (5,000 samples). This method does not rely on the assumption of normal distribution and is suitable for the skewed distribution characteristics of indirect effects, providing more robust statistical inference. When the confidence interval does not include zero, the indirect effect is considered statistically significant.

## 4 Results

### 4.1 Common method bias analysis

The correlation data were subjected to Harman’s one-way test, the KMO test, and Bartlett’s test of sphericity, with a KMO value of 0.965, Bartlett’s value of 10648.317, and degrees of freedom of 528, with a *p*-value of <0.001. These results suggest that a factor analysis is favored. Exploratory factor analysis identified 13 factors greater than 1, which together explained 38.63% (<40%) of the maximum factor variance.

To assess potential multicollinearity between variables, depression was standardized as the dependent variable, and physical activity, mindfulness, and life satisfaction were used as independent variables for covariate diagnosis. (0.776, 0.752, and 0.780), exceeding 0.1. Variance inflation factor (VIF) values (1.289, 1.330, and 1.282) were <5. Therefore, it can be inferred that there is no problem of multicollinearity in the data in the present study and that the test for mediating effects can be further conducted.

### 4.2 Correlation analysis of physical activity, mindfulness, life satisfaction and depression

According to the findings in Table 2, physical activity is significantly positively correlated with mindfulness and life satisfaction. Depression is significantly negatively correlated with physical activity, mindfulness, and life satisfaction. This implies that mindfulness and life satisfaction may have an important role in the relationship between physical activity and depression. This also provides a basis for further research into their mediating role. In terms of model fit indices, chi-square and root mean square error of approximation (RMSEA) values and comparative fit indices (CFI) were reported (*df* = 1.136, RMSEA = 0.023, CFI = 0.991, and GFI = 0.816), suggesting that the model was well fitted and suitable for further mediation analysis.

**Table 2:** Correlation analysis.

Variables	Mean (*M*)	Standard Deviation	1	2	3	4
		(*SD*)				
Physical activity	42.10	30.92	1			
Mindfulness	3.69	0.87	0.416^**^	1		
Life satisfaction	3.76	0.88	0.377^**^	0.410^**^	1	
Depression	20.65	7.77	−0.354^**^	−0.424^**^	−0.381^**^	1

^**^p<0.01.

### 4.3 Parallel mediation analysis of mindfulness and life satisfaction

The analysis of mediation effects was conducted through the Bootstrap plugin using Model 4 in Process 4.0, an SPSS macro plugin designed by Hayes again, which was specifically designed to test mediation effects. The independent variables in the study were physical activity, mindfulness, and life satisfaction as mediator variables, depression as a dependent variables and gender and age as control variables. In this study, the replicate sample was 5,000, and the default confidence interval was 95% (61). The results of regression analysis are shown in Table 3. First, physical activity showed a direct positive correlation with mindfulness and life satisfaction. Physical activity showed a negative correlation with depression (*β* = −0.0045, *p* < 0.001), which verified Hypothesis 1: Physical activity can negatively affect depression. Second, the report showed a negative relationship between mindfulness and depression (*β* = −0.2692, *p* < 0.001) and life satisfaction and depression (*β* = −0.2043, *p* < 0.001).

**Table 3:** Regression analysis of parallel mediation model of physical activity on depression.

Outcome variables	Predictor variables	Overall fit index				Significance of regression coefficients
		*R*	*R^2^*	*F*	*β*	*t*
Mindfulness	Physical activity	0.4161	0.1731	105.9263	0.0117	10.2921^***^
Life satisfaction	Physical activity	0.3774	0.1424	84.0246	0.0108	9.1665^***^
	Mindfulness					
Depression	Physical activity	0.5018	0.2518	56.5441	−0.0045	−3.7059^***^
	Mindfulness				−0.2692	−6.0892^***^
	Life satisfaction				−0.2043	−4.7955^***^

^***^p<0.001.

As shown in Table 4, the results of the mediation analysis indicate that the mediation effect is 53.54%, meaning that 53.54% of the total effect is explained by the mediating path. Furthermore, the 95% confidence interval CI (−0.0067, −0.004) does not include 0, indicating that the mediating model between physical activity and depression is valid. Second, there are three paths of influence in the mechanism of the relationship between physical activity on depression. Among them, Path 1: the direct effect of physical activity on depression (*β* = −0.0053); Path 2: the mediating role of mindfulness between physical activity and depression (*β* = −0.0031, with an effect share of 31.31%); Path 3: the mediating role of life satisfaction between physical activity and depression (*β* = −0.0022, with an effect share of 22.22%). The 95% confidence intervals of the above three paths were not 0, indicating that physical activity, mindfulness, and life satisfaction can independently affect depression. Meanwhile, mindfulness and life satisfaction showed parallel mediating roles in the relationship between physical activity and college students’ depression, which verified hypothesis H2: mindfulness mediates the relationship between physical activity and depression. As well as verified hypothesis H3: life satisfaction mediates the relationship between physical activity and depression. Finally, we compared the indirect effect differences between the two paths, and the results were not significant. This also means that there are two mediating paths in the model, and the parallel mediation model is valid.

**Table 4:** Parallel mediation tests of mindfulness and life satisfaction in physical activity on depression.

Related Paths	Effect	BootSE	BootLLCI	BootULCI	Measures of intermediary effect
Total indirect effect	−0.0053	0.0007	−0.0067	−0.004	53.54%
Path 1: Physical Activity → Mindfulness → Depression	−0.0031	0.0006	−0.0044	−0.002	31.31%
Path 2: Physical activity → Life Satisfaction → Depression	−0.0022	0.0005	−0.0032	−0.0013	22.22%
Contrasting indirect effects (P1-P2)	−0.0009	0.0009	−0.0026	0.0007	

### 4.4 Moderating effect of gender

A moderated mediation effect test was conducted through Model 7 of PROCESS. The results showed that the physical activity and gender interaction predicting depression were not significant after controlling for grade level and education. Subsequently, mediation effects under the different moderated variables were further analyzed, and none of the results were significant. Thus, the results indicated that there was no significant moderate mediation effect, meaning that there was no significant gender difference in the mediating effect of mindfulness and life satisfaction.

## 5 Discussion

**Figure 2:**
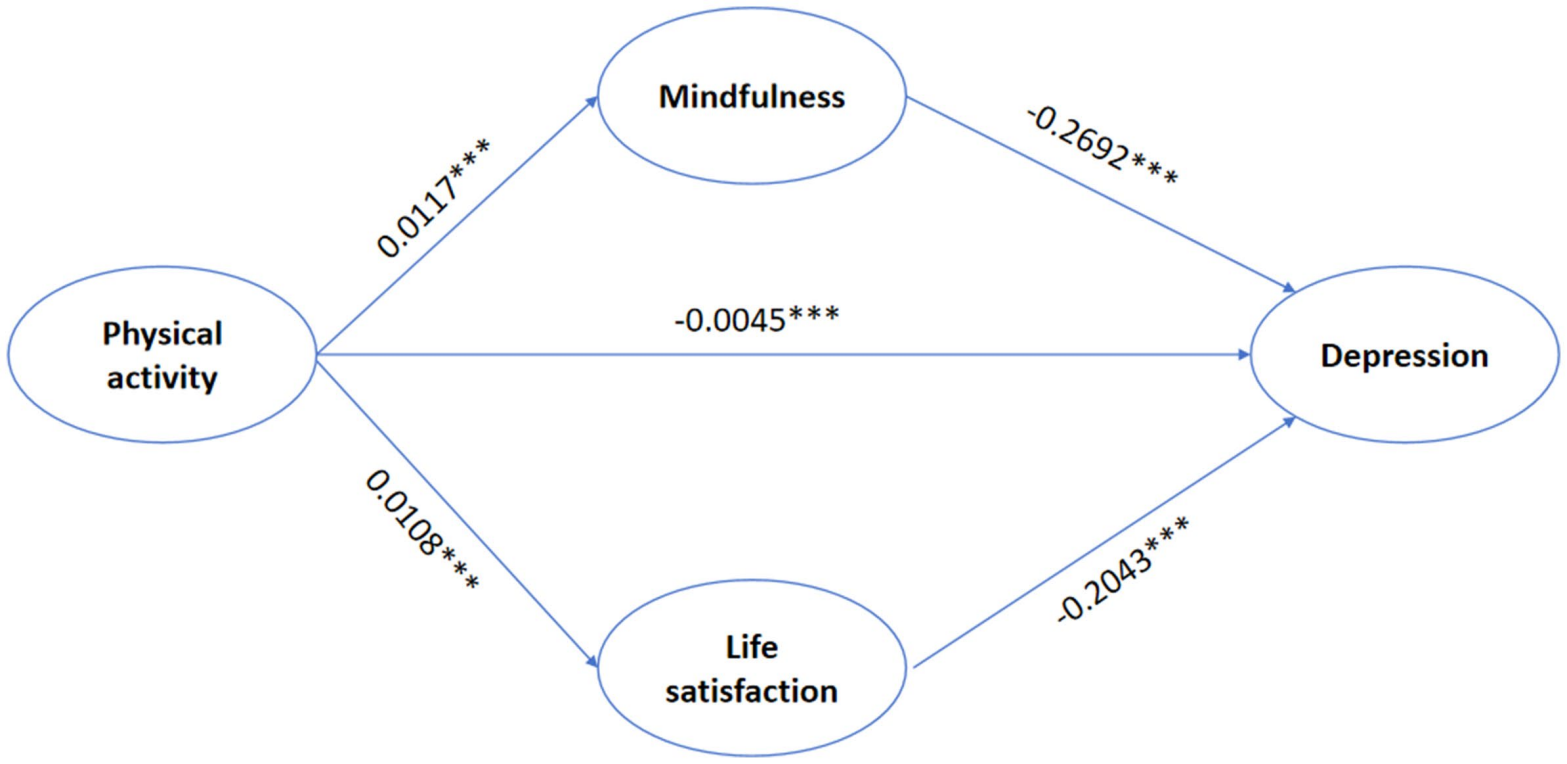
Parallel mediation model of mindfulness and life satisfaction in physical activity on depression ^***^p<0.001.

This study examined the role of mindfulness and life satisfaction in the relationship of depression among college students in North China, as well as the relationship of physical activity to the depression level of college students, which verified Hypotheses 1, 2, and 3. In addition, this study also explained that college students reduced their depression level through physical activity, thereby enhancing mindfulness and life satisfaction, which further provides a scientific basis for the design of future intervention programs to alleviate college students’ mental health (Figure 2) design provides a scientific basis.

### 5.1 Direct effect of physical activity on depression

The results of this study indicate that college students’ level of physical activity can have a negative effect on their own depression. This finding validates Hypothesis 1. This is consistent with the study by Jelleli et al. (62), which provides further validation of the mechanism of alleviating depression through participation in physical activity. The negative predictive effect of physical activity on depression among college students remained significant after the inclusion of mediating variables. According to Svensson et al., regular physical activity has been shown to have a positive effect on levels of depression (63). The neural hypothesis suggests that participation in physical activity stimulates the release of endorphins, chemicals in the brain that promote feelings of well-being and relaxation, as well as decreasing levels of stress hormones such as cortisol (64). Participation in physical activity can also be an effective way to manage stress and negative emotions (65, 66). Many studies have found an association between physical activity and the quality of mental health (67). And emphasized that mental health status is related to physical inactivity. According to self-determination theory, individuals who maintain the habit of engaging in physical activity, achieving exercise goals, and accomplishing corresponding exercise challenges can provide themselves with psychological buffers and enhance positive emotional experiences (68). Synthesizing the findings of this study and previous research, this study concluded that physical activity can effectively alleviate depression and promote the development of college students’ psychological health.

### 5.2 Mediating role of mindfulness

Building on the findings regarding the direct effects of physical activity on depression, this study further explored the potential mediating role of mindfulness in this relationship to gain a deeper understanding of how physical activity influences an individual’s level of depression. In the path analysis of the relationship between physical activity and depression, mindfulness was found to have a significant mediating effect, thereby validating Hypothesis 2. Previous studies have shown that mindfulness can regulate emotions and promote mental health, and it is also effective in alleviating negative emotions such as depression, anxiety, and stress (69, 70). In addition, mindfulness permits the acquisition of certain protective factors that enhance psychological functioning in situations of intense stress. Thus, individuals with high levels of mindfulness will have higher cognitive resilience, psychological well-being, emotional balance, etc., which will reduce their anxiety, depression, and burnout (71). Meanwhile, the differences between college students with different education, grades, and gender on mindfulness and depression during physical activity were statistically significant. In addition, in this study, physical activity was found to be effective in promoting the increase of mindfulness level of college students, and by increasing the level of mindfulness, reduce the risk of depression. This is like previous studies that physical activity has a significant positive predictive effect on mindfulness and that there is a negative correlation between physical activity and mindfulness on individual depression (34, 72). Based on this, the present study examined the role of physical activity in reducing depression by enhancing mindfulness among college students. Maintaining healthy physical activity habits will help college students enhance their mindfulness and reduce their depression.

### 5.3 Mediating role of life satisfaction

In addition to mindfulness, this study also examined the role of life satisfaction as another potential mediating variable, aiming to capture broader emotional and cognitive assessment dimensions in the relationship between physical activity and depression. Path analysis using life satisfaction as a mediating model showed that life satisfaction mediated the relationship between physical activity and depression and had a significant effect. According to the mediation effect test, physical activity not only negatively predicts depression among college students but also affects the level of depression among college students by increasing their life satisfaction profile. This finding validates Hypothesis 3. In addition, the symptoms of depressed mood partially explain the level of relationship between physical activity and life satisfaction, and the extent of this association shows that a high degree of depressed mood may be caused by an individual’s lack of physical activity and result in a low level of life satisfaction. It has been shown that life satisfaction is negatively correlated with anxiety. The higher the life satisfaction, the lower the anxiety level of an individual’s anxiety level (61). Individuals with higher levels of life satisfaction choose to live more active lifestyles and adopt more positive health behaviors and coping strategies when dealing with mental health problems such as anxiety. Research has shown that individuals’ levels of life satisfaction are related to their quality of life, and higher levels of life satisfaction are associated with more effective coping with negative emotions, which in turn enhances individuals’ quality of life (73). Given the intrinsic link and mediating effect of life satisfaction between physical activity and depression, college students can positively influence their life satisfaction through physical activity, thus reducing their depression.

### 5.4 Parallel mediating role of mindfulness and life satisfaction

In summary, the findings suggest a strong correlation between physical activity and depression. To obtain a more comprehensive understanding, the present study further constructed a parallel mediation model with both positive thoughts and life satisfaction as two variables to examine their parallel mediating effects between physical activity and depression. The analysis of the parallel mediation model between sport and depression using mindfulness and life satisfaction as mediating variables showed that the two could not only present mediating effects individually but also play parallel mediating roles in the model. This is consistent with the theory proposed by previous researchers that increased levels of mindfulness and life satisfaction are effective in alleviating depression, stress, and anxiety (24). The current study provides further support for this and suggests potential generalizability in specific populations. The purpose of physical activity is to promote a lifelong healthy lifestyle (74). From this perspective, the current results suggest that physical activity promotes levels of mindfulness and life satisfaction, further helping to reduce the emergence of depression in college students. This interpretation resonates with mind–body monism (75), which posits that the body and mind are essentially an inseparable whole, together constituting the unified existence of a person. In other words, participating in sports activities not only enhances mindfulness levels but is also closely related to an individual’s mental health. Synthesizing the results of this study shows that the higher an individual’s level of mindfulness and life satisfaction, the more positive and healthier a response to future events will be adopted, thus contributing to the reduction of depression. Integrating physical activity to enhance mindfulness and life satisfaction will provide a more comprehensive framework for improving an individual’s level of depression.

## 6 Research limitations and future perspectives

The main contribution of this study lies in further elucidating the mechanisms underlying the relationship between physical activity and depression among Chinese college students, and revealing the key role of mindfulness and life satisfaction in this relationship, thereby enriching the existing research on the association between physical activity participation and depression among college students. However, this study still has some limitations.

First, the cross-sectional design of this study limits the ability to infer causal relationships between physical activity and depression, as cross-sectional designs cannot directly establish causal relationships. Future researchers may validate the mediating effects of mindfulness and life satisfaction through longitudinal studies and further consider the influence of differences in college students’ majors and places of residence.

Second, due to data and questionnaire limitations, this study selected life satisfaction and mindfulness as mediating variables, which may introduce bias in the results. Future researchers could validate the findings of this study by testing the scales of mindfulness and life satisfaction, including conducting further psychometric validation.

Finally, this study only considered two mediating variables. There may be other mediating variables between physical activity and depression. Future research could consider incorporating more specific mediating variables, such as self-efficacy, resilience, and subjective well-being. These variables have a solid theoretical foundation and empirical support in cognitive behavioral theory, positive psychology, and health psychology, and therefore have high research expansion potential.

## 7 Conclusion

The results of the study indicate that physical activity has direct and indirect effects on depression in college students. Physical activity indirectly affects depression by influencing mindfulness and life satisfaction. At the same time, physical activity can increase college students’ life satisfaction and thus alleviate depressive symptoms. In today’s world, where individual physical and mental health is emphasized, these findings remind society and educational institutions to pay attention to the physical and mental health of the college student population. In addition, this study further reveals the different sources of depression among college students in the context of Chinese society, as well as the different factors that influence the occurrence of depression. In the future, further enhancement of college students’ participation in physical activities can promote their mental health to a considerable extent.

## Data Availability

The original contributions presented in the study are included in the article/Supplementary material, further inquiries can be directed to the corresponding author.
